# Repetitive Behaviors in Autism and Obsessive-Compulsive Disorder: A Systematic Review

**DOI:** 10.1007/s10803-024-06357-8

**Published:** 2024-04-23

**Authors:** Jessica O’Loghlen, Matthew McKenzie, Cathryne Lang, Jessica Paynter

**Affiliations:** 1 School of Applied Psychology, Griffith University, 58 Parklands Drive, Southport, QLD 4215 Australia; 2 Building N23, -1.03, 170 Kessels Road, Nathan, QLD 4111 Australia

**Keywords:** ASD, OCD, Differential diagnosis, Dual diagnosis, Comorbidity, Compulsions

## Abstract

Obsessive-compulsive disorder (OCD) and autism are characterized by the presence of repetitive behaviors. Differentiating between repetitive behaviors attributable to a diagnosis of autism, and those attributable to OCD, poses challenges for differential and co-occurring diagnosis. Differentiation is important to inform appropriate supports and interventions for phenotypically similar but functionally distinct behaviors. In this systematic review, the quantitative literature was examined to explore the similarities and differences in repetitive behaviors (including restricted and repetitive behaviors and interests, and obsessive-compulsive behaviors) in autistic individuals and those with OCD, and those with co-occurring diagnoses, in terms of: (1) expression, (2) content, and (3) associated factors. Methods: Thirty-one studies were identified that compared repetitive behaviors in autistic individuals, individuals with OCD, or individuals with both diagnoses. Results: The results suggest considerable overlap in the intensity and content of repetitive behaviors between groups. The findings of this review highlight that research aimed specifically at understanding similarities and differences in repetitive behaviors between autistic individuals and individuals with OCD is limited and frequently only compare at total score or composite measure levels. Conclusion: Further research into differences in the presentation of repetitive behaviors at a subscale and item level is required to inform clearer differentiation of specific behaviors in autism versus OCD. Understanding and more accurately differentiating is essential for efficient diagnosis, effective treatment, and better outcomes.

## Introduction

Autism and obsessive-compulsive disorder (OCD) diagnostic criteria each include forms of restricted or repetitive behavior (American Psychiatric Association [APA], 2022). For autistic individuals, this includes stereotyped movements, ritualized patterns of verbal/nonverbal behavior, and restricted interests. For people with OCD, this includes compulsions related to cleaning, checking, ordering, or arranging (APA, 2022). These repetitive behaviors (henceforth used to include restricted and repetitive behaviors and interests [RRBIs] and obsessive-compulsive behaviors) can appear similar, e.g., repeated recital of verbal information such as a movie script in autism or repetitive chanting in OCD. Autism frequently co-occurs with psychiatric conditions including OCD (> 17.4%; van Steensel et al., 2011) and autism diagnoses are often un/under-detected in individuals with OCD, even for those receiving clinical support (e.g., Wikramanayake et al., 2018). Considering the high rates of co-occurrence and phenotypical similarities, clinicians likely encounter presentations of repetitive behavior that do not clearly correspond to one diagnosis over the other.

Two review papers have begun to explore differences in repetitive behavior in autism and OCD. In a narrative review, Paula-Pérez (2013) posited that the emotional valence attached to engagement in repetitive behaviors was a key feature to inform differential diagnosis, based on the ego-syntonic (i.e., expressions harmonious with self-concept and goals, without sparking heightened anguish or self-recrimination) experience of RRBIs for autistic people versus the ego-dystonic (i.e., expressions inconsistent with self-concept and goals and accompanied by an increase in anguish or self-recrimination) experience of compulsions in people with OCD. Dystonic experiences were also described by the sensation that the content of the thought or behavior was foreign, and out of the control of the person experiencing it, which aligns with experiences of engaging in compulsive behavior reported by individuals with OCD (Keyes et al., 2018). This review highlights potential internal differences between repetitive behaviors characteristic of autism versus OCD, noting emotional valence differences, despite similar observable behaviors.

In a comparative review, Jiujias et al. (2017) posited that repetitive behaviors could be differentiated between autistic individuals and individuals with OCD based on differences in associations with anxiety, executive functioning (EF), and sensory processing. They hypothesized that while anxiety has a bidirectional role in OCD (i.e., obsessions produce anxiety and compulsions alleviate anxiety), the directionality of the relationship between anxiety and RRBIs in autism is unclear (i.e., does anxiety perpetuate RRBIs, or do RRBIs relieve anxiety? ). They also found different relationships between EF and repetitive behaviors between conditions. Poor inhibitory control (i.e., the ability to control and manage thoughts and impulses) was associated with repetitive behaviors in OCD, and difficulties with set-shifting (i.e., the ability to shift attention between one task and another) were associated with repetitive behaviors in autism. Finally, they acknowledged differences in sensory processing as contributing to repetitive behaviors in both conditions based on emerging literature showing associations between sensory integration difficulties, obsessions, and an increased need for control. This review highlights important distinctions and potential overlaps between RRBIs and obsessive-compulsive behaviors, including shared associations with sensory processing, which is widely acknowledged in autism, but less commonly recognized in OCD.

While these reviews provide an important starting point, the absence of a systematic approach to either review limits the degree to which these interpretations can be considered representative of the extant literature, which is essential for informing clearer parameters for differential diagnosis, as well as meaningful directions for future research. Further, the most recent review (Jiujias et al., 2017) was published in 2017, since which time many more studies have been published (e.g., Dingemans et al., 2022; Kushki et al., 2019). Thus, the aim of this systematic review is to examine the similarities and differences in repetitive behaviors in autistic individuals and individuals with OCD in terms of: (1) expression, (2) content, and (3) associated factors (e.g., anxiety).

## Method

This review was conducted following the Preferred Reporting for Items for Systematic Reviews and Meta-Analyses (PRISMA) statement (Page et al., 2021) with quantitative components informed by Pickering and Byrne (2014). The review was pre-registered on PROSPERO (Registration No. CRD42022351325) with a minor variation (12/07/2023) added that eligible studies needed to include both a quantitative measure of repetitive behavior and a comparison between diagnostic groups, leading to the exclusion of studies which did not report an explicit comparison (e.g., Zandt et al., 2009).

## Search Strategy

Search terms were grouped by target population (i.e., autism and OCD) and behavior of interest (i.e., RRBIs and compulsive behaviors) (see Appendix A). Electronic database searches were conducted in five databases: Science Direct, PsycINFO, Web of Science, Scopus, and CINAHL. All searches were performed by the first author (JO). The initial search was executed on 18 August 2022. An updated search was executed on 30 June 2023.

### Review Criteria

#### Participants

Eligible sample participants were individuals of any age with a diagnosis of autism and/or a diagnosis of OCD, and pattern(s) of restricted and repetitive behaviors and interests, and/or compulsive behaviors.

#### Comparator

Studies were required to include at least two groups that could comprise autism-only compared to OCD-only, OCD-only compared to autism + OCD, or autism-only compared to autism + OCD, and include a measure of repetitive behaviors. Studies with three or more groups (i.e. autism-only, OCD-only, and autism + OCD) were also included.

#### Eligibility Criteria

Quantitative studies of all designs were considered. This included non-randomized studies of interventions and randomized control trials. For intervention studies, only measures of repetitive behavior collected at baseline were included, because the interventions implemented may have influenced group differences. Inclusion criteria were studies that: (1) included participants of any age with a diagnosis of autism, (2) included a comparison group of any age with a diagnosis of OCD (including co-occurring autism and OCD), (3) included a quantitative measure of RRBIs, and/or compulsive behaviors, and/or OCD symptomology, and a comparison of this measure between diagnostic groups (i.e., between autistic individuals and individuals with OCD, or either group and a comparison group with both conditions), (4) were peer-reviewed, (5) were full-text, and (6) were written in English. Diagnosis of autism included Autism Spectrum Disorder (ASD, e.g., DSM-5-TR; APA, 2022), and earlier diagnoses made using previous diagnostic criteria (e.g., DSM-IV; APA, 1994) or other diagnostic systems (e.g., ICD-10; WHO, 2004) including Autistic Disorder, Asperger’s Syndrome, and Pervasive Developmental Disorder – Not Otherwise Specified. Diagnoses included in earlier classifications of Pervasive Developmental Disorders (i.e., Rett Syndrome, Childhood Disintegrative Disorder; APA, 1994), but not included under the current autism classification (APA, 2022), were excluded.

Papers including participants with other additional co-occurring conditions were also considered for inclusion, given the high rates of co-occurring conditions observed in autistic individuals (APA, 2022). Exclusion criteria were studies that involved: (1) non-human research (e.g., mice models), (2) participants without diagnoses (e.g., traits only), (3) a single clinical population only (i.e., with no comparison clinical group), (4) an autism or OCD population not separately reported in a study evaluating a range of diagnoses, and/or (4) those that did not include a quantitative measure of repetitive behaviors. Qualitative studies were also excluded. In the absence of a comparable previous systematic review, no date restrictions were placed on the searches.

#### Study Selection and Coding

**Fig. 1:**
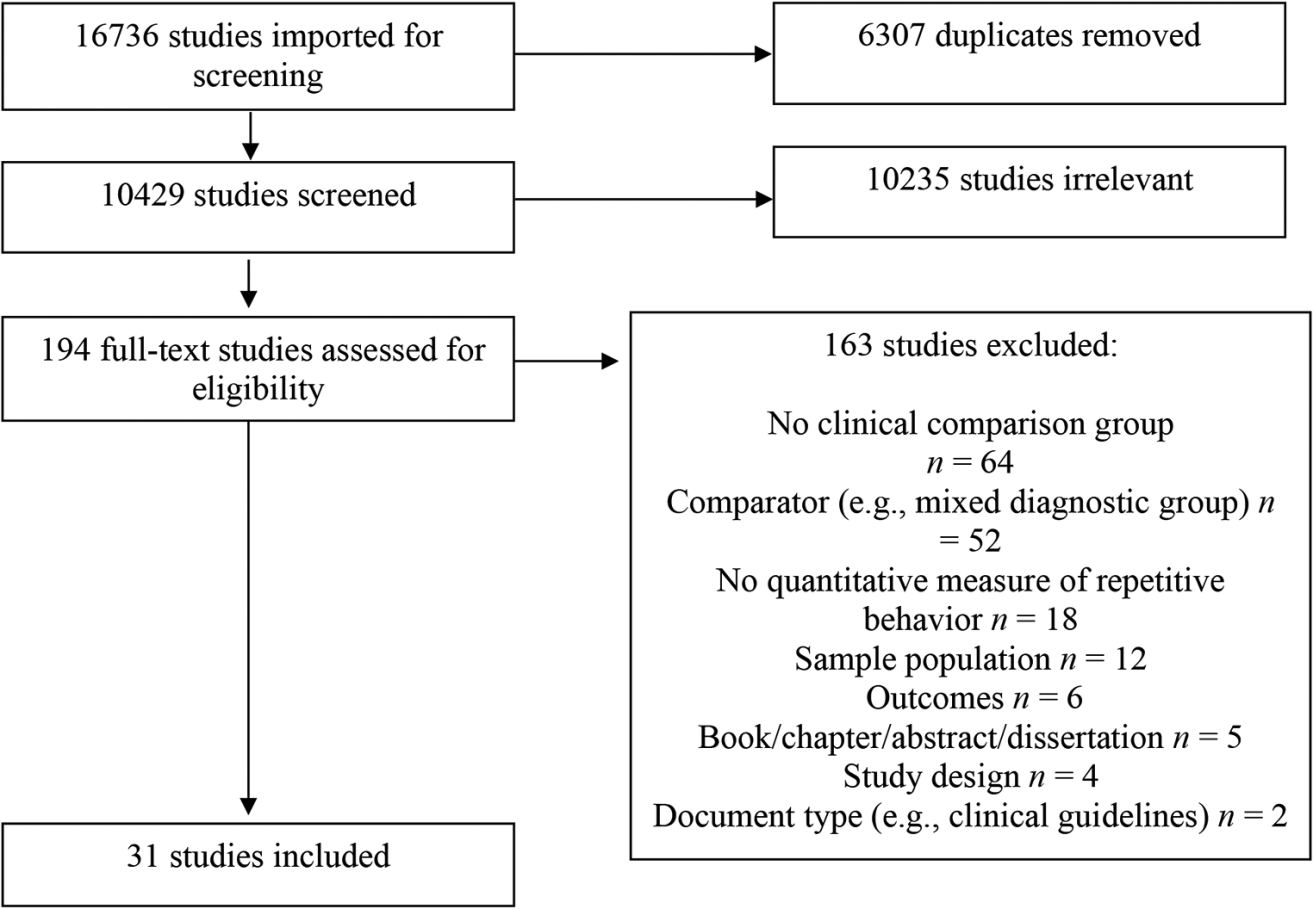
PRISMA flow diagram

The screening process comprised: (1) removal of duplicates; (2) title and abstract screening; and (3) full text screening and data charting (Fig. 1). Covidence was used to manage the screening process. Database searches (August 2022; June 2023) yielded 16,736 papers, including 6307 duplicates. Following de-duplication, the remaining 10,429 papers were screened by title and abstract by the first author. A second reviewer screened 20% of titles and abstracts (*n* = 2086) against the inclusion and exclusion criteria. Papers were sorted into exclusions, inclusions, or assigned for full text review if unclear based on the title/abstract. Initial agreement between reviewers was substantial (Cohen’s κ = 0.85), with 100% agreement following discussion. A total of 194 papers were then reviewed in full text. These remaining papers were reviewed by the first author, with 20% checked by a second reviewer (*n* = 39). There was 100% agreement on papers meeting the inclusion and exclusion criteria. This review resulted in 31 papers deemed eligible for inclusion, and data from these was extracted using a template in Covidence. A sample of 20% were also extracted by a second independent reviewer and checked to confirm reliability, with 100% agreement on the extracted data.

#### Data Extraction

Data extracted from each full text record included title, author(s), year of publication, country of origin, objective(s), design and methodology, community involvement in the research (i.e., input from autistic individuals or individuals with OCD), participant demographics (age, gender, ethnicity, IQ/cognitive ability, and communication/language ability), diagnostic characteristics (including diagnostic tool/method used to ascertain autism and/or OCD diagnosis), presence of other co-occurring conditions (e.g., anxiety disorders), inclusion/exclusion criteria, method(s) of data collection, RRBIs/obsessive-compulsive behavior(s) measured (including measure), level of measurement (total score only, subscale scores, or item-level analyses), presence/level of repetitive behaviors by group (mean, standard deviation), reported experience of repetitive behaviors (if reported), content/focus of repetitive behaviors (e.g., special interests), covariates assessed (e.g., anxiety), functional assessment/motivation behind repetitive behaviors (if reported), statistical analyses used, statistical results of group comparisons, correlations/interactions with covariates, and key findings. Where possible, findings based on specific sub-categories of behavior were also extracted such as subscale scores and individual item scores.

#### Quality Assessment

Quality assessment was conducted by the first author, with a sample of 20% conducted by a second independent reviewer. The quality of included studies was appraised using published guidelines by Hawker et al. (2002). These guidelines were selected due to their suitability for assessing methodological rigor across studies using different research designs. The methodological rigor of each aspect of the study (e.g., theoretical framework, sampling, approach to and explanation of data analysis) and the generalizability and implications of the findings is ranked from (1) Very Poor to (4) Good, based on established individual criteria, and these scores are summed to indicate the quality of each study (i.e., overall scores of < 10 = Very Poor to 40 = Very Good; Hawker et al., 2002). Initially, there was 89% agreement between reviewers. Following discussion of seven discrepancies, 100% agreement was achieved. Resolution of these discrepancies did not impact on the final rating of any study. A web-based AMSTAR 2 (A [revised] MeaSurement Tool to Assess systematic Reviews; Shea et al., 2017) checklist was also used to assess the quality and reliability of the systematic review as a whole.

#### Data Synthesis

Data were synthesized through quantitative and narrative synthesis. To address the review questions, data pertaining to specific sub-categories of behavior were critically compared. Categories of behavior were determined based on the measure used to assess repetitive behaviors across the included studies. For example, for studies including a subscale analysis of RRBIs using the Repetitive Behavior Scale – Revised (RBS-R; Bodfish et al., 2000), these sub-categories were differentiated as: (1) stereotyped behavior, (2) self-injurious behavior, (3) compulsive behavior, (4) routine behavior, (5) sameness behavior, and (6) restricted behavior. Sub-categorization of OCD behaviors was made in line with recognized OCD symptom dimensions, for example, based on the Y-BOCS Symptom Checklist (i.e., cleaning/washing, checking, repeating, counting, ordering/arranging, hoarding, and miscellaneous compulsions; Storch et al., 2010).

## Results

### Overview of Included Studies

A total of 31 studies were included from the United Kingdom (*n* = 5; 16%), the Netherlands (*n* = 4; 13%), the United States (*n* = 4; 13%), Japan (*n* = 3; 10%), Italy (*n* = 2; 6%), Australia (*n* = 2; 6%), Canada (*n* = 2; 6%), Norway (*n* = 1; 3%), Sweden (*n* = 1; 3%), and France (*n* = 1; 3%). Six studies (19%) were conducted across multiple countries including data from the Netherlands, the United Kingdom, Germany, and Sweden. Most employed a cross-sectional (*n* = 9; 29%) or case control (*n* = 9; 29%) design. Appendix B outlines the key characteristics of each included study.

Demographic data for the total sample are provided in Table 1. In the 31 included studies, 1123 autistic participants, 982 participants with OCD, and 262 participants with both conditions (i.e., autism and OCD) were included. Only four studies reported participants’ ethnicity (Akkermans et al., 2019; Hollestein et al., 2021; Lewin et al., 2011; Pertusa et al., 2012). Of the 253 participants represented across these four studies, 231 (91.3%) were White. Only two studies (6.3%) included participants with a below average intelligence or intellectual impairment, with most studies (*n* = 23; 74%) excluding participants on the basis of below average intelligence, co-occurring intellectual or developmental conditions, or language impairment. Sixteen studies (52%) examined repetitive behaviors in children or adolescents and fourteen studies (45%) examined repetitive behaviors in adults. One study (3%) included both adolescent and adult participants.

**Table 1:** Demographics of autistic participants, participants with OCD, and participants with both diagnoses

Demographic	No. of studies where reported (for subgroups) *n*	**Autism** (*N* = 1123)	OCD (*N* = 982)	Autism + OCD (*n* = 262)
		*n* (%)		
**Age group**	31 (*n* = 2367)			
Children		194 (17.3%)	130 (13.2%)	35 (13.4%)
Children/adolescents		272 (24.2%)	232 (23.6%)	60 (22.9%)
Adolescents/adults		73 (6.5%)	53 (5.4%)	0
Adults		584 (52.0%)	567 (57.7%)	167 (63.7%)
**Gender**	30 (*n* = 2175)	1109	866†	200†
Male		820 (73.9%)	424 (49.0%)	130 (65.0%)
Female		289 (26.1%)	442 (51.0%)	70 (35.0%)
**Co-occurring conditions**	13 (*n* = 422)‡			
Attention-deficit hyperactivity disorder		35 (17.6%)	11 (8.5%)	8 (8.5%)
Anxiety		34 (17.1%)	20 (15.5%)	11 (11.7%)
Mood disorders		50 (25.1%)	36 (27.9%)	24 (25.5%)
Panic/agoraphobia		14 (7.0%)	6 (4.7%)	0
Tics		3 (1.5%)	28 (21.7%)	25 (26.6%)
Tourette’s syndrome		0	1 (0.8%)	2 (2.1%)
Dysthymia		9 (4.5%)	2 (1.6%)	1 (1.1%)
Social phobia		14 (7.0%)	12 (9.3%)	10 (10.6%)
Social anxiety disorder		7 (3.5%)	1 (0.8%)	2 (2.1%)
Specific phobia		0	5 (3.9%)	6 (6.4%)
Separation anxiety		0	2 (1.6%)	3 (3.2%)
Oppositional defiant disorder		11 (5.5%)	1 (0.8%)	3 (3.2%)
Conduct disorder		1 (0.5%)	0	0
Eating disorders		3 (1.5%)	2 (1.6%)	0
Post-traumatic stress disorder		5 (2.5%)	0	1 (1.1%)
Schizophrenia		4 (2.0%)	0	0
Psychosis		9 (4.5%)	0	0

†Missing data from one study (Wikramanayake et al., 2018)

‡Autism *n* = 199; OCD *n* = 129; Autism + OCD *n* = 94

### Quality of Included Studies

The quality of most included studies (*n* = 29; 94%) ranged between good and very good, except for two studies which were rated as fair-poor (*n* = 1; 3%) and poor-very poor (*n* = 1; 3%) (for full scoring see Appendix C). No studies reported explicit input from autistic individuals or individuals with OCD.

### Quality of the Systematic Review

A high level of confidence in the results of this review was indicated based on a critical appraisal via AMSTAR 2, suggesting that the current review provides an accurate and comprehensive summary of the results of the available studies that address the question of interest (Shea et al., 2017; Appendix D).

### Expression of Repetitive Behaviors across Groups

Studies used a variety of different standardized measures to assess the expression of repetitive behavior. Some measures reported on the intensity or severity of repetitive behavioral presentations (e.g., Y-BOCS) whereas others reported on the frequency or breadth of repetitive behaviors endorsed (e.g., RBS-R and Repetitive Behavior Questionnaire [RBQ; Turner, 1995]). Thus, expression of repetitive behaviors in the following analyses encompasses both the frequency and/or intensity of repetitive behavioral presentations.

### Expression of RRBIs

Relatively consistent findings were observed when comparing autism-related RRBIs between groups. The majority of studies (91% of studies making this comparison) found no significant differences in the overall intensity of RRBIs between autistic individuals, individuals with OCD, and individuals with both conditions (Akkermans et al., 2019; Cadman et al., 2015; DiCriscio et al., 2016; Gooskens et al., 2019, 2021; Hollestein et al., 2021; Jacobs et al., 2021; Lamothe et al., 2022; Naaijen et al., 2017; Zandt et al., 2007). In contrast, a single study (Sturm et al., 2018) found autistic children to have more intense levels of RRBIs compared to children with OCD. However, these differences may be related to the measure used, with differences in the endorsement of RRBIs only found when using the Social Responsiveness Scale (SRS; *restricted interests and repetitive behavior* subscale; Constantino & Gruber, 2012) in this study, but not the RBS-R or RBQ in other studies (e.g., Akkermans et al., 2019). Three studies also compared intensity of compulsive behaviors as a sub-construct of RRBIs between autistic children and children with OCD. Two studies found that children with OCD reported significantly greater compulsivity (Gooskens et al., 2019, 2021), whereas another study found that autistic children reported significantly greater compulsivity (Naaijen et al., 2017).

### Expression of Compulsions

When comparing individuals with a single diagnosis, findings in relation to OCD-related compulsions were relatively consistent. Six studies (86% of studies making this comparison) reported individuals with OCD had significantly greater total obsessive-compulsive symptom severity compared to autistic individuals (Bejerot et al., 2014; DiCriscio et al., 2016; Kushki et al., 2019; Russell et al., 2005; Ruta et al., 2010; Zandt et al., 2007; also Niemeyer et al., 2022 though significance was not reported). Three studies also reported that children with OCD had significantly greater compulsive symptom severity at the subscale level, than autistic children (DiCriscio et al., 2016; Kushki et al., 2019; Zandt et al., 2007). Significantly greater total obsessive-compulsive symptom severity in individuals with OCD was reported using a variety of different measures including the Brief Obsessive-Compulsive Scale (BOCS; Bejerot et al., 2014), Toronto Obsessive-Compulsive Scale (TOCS; Park et al., 2016) (Kushki et al., 2019), and CY-BOCS/Y-BOCS (DiCriscio et al., 2016; Russell et al., 2005; Ruta et al., 2010; Zandt et al., 2007). Exceptions were Dingemans et al. (2022) who reported no significant differences in obsessive-compulsive symptom severity between autistic individuals and individuals with OCD via the Padua Inventory – Revised (Sanavio, 1988), Mack et al., (2010) who reported no significant differences in impairment associated with obsessive-compulsive symptoms between children with OCD and autistic children with co-occurring OCD via the Children’s Obsessive Compulsive Inventory (ChOCI; Shafran et al., 2003).

The majority of studies (67% of studies making this comparison) comparing individuals with OCD with individuals with both conditions found no significant differences in total obsessive-compulsive symptom severity (Griffiths et al., 2017; Lewin et al., 2011; Mack et al., 2010; Mito et al., 2014; Murray et al., 2015; Nakagawa et al., 2019; Tsuchiyagaito et al., 2017; Wikramanayake et al., 2018). Studies that did report differences between these groups found that individuals with both conditions reported significantly greater symptom severity than autistic individuals (Helverschou et al., 2009; Lamothe et al., 2022; Niemeyer et al., 2022), except for Cath et al. (2008) who found that adults with OCD reported significantly greater total symptom severity (but not compulsive symptom severity at the subscale level) compared to autistic adults with co-occurring OCD.

### Content of Repetitive Behaviors across Groups

#### Content of RRBIs

Studies examining differences in the content of autism-related RRBIs were limited, with mixed results. Hollestein et al. (2021) found no significant differences in the intensity of stereotypy, compulsivity, ritualistic behaviors, or limited interests between children with OCD and autistic children. However, in the same study, autistic children reported significantly greater insistence on sameness behaviors compared to children with OCD at the first time point. One year later, children with OCD reported significantly more insistence on sameness behaviors than autistic children. Similarly, Zandt et al. (2007) found that children with OCD were rated similarly to autistic children in their intensity of most types of RRBIs (repetitive language, stereotyped movement, self-injury, manipulation of objects, sameness environment, attachment to objects, limited interests), except for routines and rituals, where children with OCD reported greater intensity of routine and ritual behaviors.

In adults, only one study examined RRBIs between groups at the symptom level. Fusar-Poli et al. (2020) examined the presence of RRBIs via two individual items on the Autism Spectrum Disorder in Adults Screening Questionnaire (ASDASQ; Nylander & Gillberg, 2001) between autistic adults and adults with OCD. Autistic adults reported more ritualized, routine- and rule-oriented behaviors, and special interests than adults with OCD.

#### Content of Compulsions

Mixed findings in relation to the content of compulsive behaviors were found, with most studies comparing the content of compulsions via the Children’s Yale-Brown Obsessive-Compulsive Scale (CY-BOCS; Goodman et al., 1991) or Y-BOCS. The frequency of counting compulsions endorsed by autistic individuals, individuals with OCD, and individuals with both conditions was generally similar (Griffiths et al., 2017; Lewin et al., 2011; Mack et al., 2010; Mito et al., 2014; Nakagawa et al., 2019; Russell et al., 2005; Wikramanayake et al., 2018). Two exceptions found conflicting results. McDougle et al. (1995) found that adults with OCD endorsed more counting compulsions than autistic adults, whereas Zandt et al. (2007) found that counting compulsions were highly endorsed by autistic children and not at all by children with OCD. The frequency of repeating compulsions endorsed was also generally similar between groups (Griffiths et al., 2017; Mack et al., 2010; McDougle et al., 1995; Mito et al., 2014; Nakagawa et al., 2019; Ruta et al., 2010; Wikramanayake et al., 2018), with some exceptions reporting more frequent endorsement of repeating compulsions by individuals with OCD compared to autistic individuals (Russell et al., 2005; Zandt et al., 2007) or individuals with both conditions (Lewin et al., 2011).

More variable findings were found for checking and hoarding compulsions. A number of studies reported greater frequencies of checking compulsions by individuals with OCD compared to autistic individuals (Bejerot et al., 2014; McDougle et al., 1995; Russell et al., 2005; Ruta et al., 2010; Zandt et al., 2007) or individuals with both conditions (Lewin et al., 2011), although others found no significant differences between groups (Griffiths et al., 2017; Mack et al., 2010; Mito et al., 2014; Nakagawa et al., 2019; Wikramanayake et al., 2018). In support of the former, others reported more severe checking compulsions by individuals with OCD (Cadman et al., 2015) or both conditions (Cadman et al., 2015; Helverschou et al., 2009) compared to autistic individuals via the Obsessive-Compulsive Inventory – Revised (OCI-R; Foa et al., 2002) and Psychopathology in Autism Checklist (Helverschou et al., 2009) respectively. In contrast, Dingemans et al. (2022) found no differences in the severity of counting compulsions between groups via the Padua Inventory – Revised. The use of different assessment measures may have contributed to differences in these findings.

Several studies also found that hoarding compulsions were endorsed in similar frequencies between groups (Griffiths et al., 2017; Lewin et al., 2011; Mack et al., 2010; Russell et al., 2005; Ruta et al., 2010; Wikramanayake et al., 2018; Zandt et al., 2007). However, some variable findings were reported amongst adults. Several studies found that hoarding compulsions were more frequently endorsed by (McDougle et al., 1995) or more intense in (Cadman et al., 2015; Pertusa et al., 2012) autistic adults compared to adults with OCD. Others found that hoarding compulsions were more frequently endorsed by (Mito et al., 2014; Nakagawa et al., 2019) or severe in (Cadman et al., 2015) adults with both conditions compared to adults with OCD only.

In many studies, the intensity (Cadman et al., 2015) or frequency (Griffiths et al., 2017; Lewin et al., 2011; Mack et al., 2010; Mito et al., 2014; Russell et al., 2005; Ruta et al., 2010; Wikramanayake et al., 2018; Zandt et al., 2007) of ordering/arranging compulsions was similar between groups. In others, adults with OCD more frequently endorsed ordering/arranging compulsions compared to autistic adults (Bejerot et al., 2014). In further contrast, others found that autistic adults (McDougle et al., 1995) or adults with both conditions (Nakagawa et al., 2019 [post-treatment]) endorsed significantly more ordering/arranging compulsions than adults with OCD only. Differences between these studies’ methodology that may explain contrasts in results including the use of different standardized measures (e.g., BOCS; Bejerot et al., 2014), the inclusion of autistic participants with cognitive impairments (McDougle et al., 1995), or the effects of a therapeutic intervention (i.e., cognitive-behavioral therapy; Nakagawa et al., 2019).

Mixed findings were also found in relation to washing/cleaning compulsions. Some studies reported no significant differences in severity (Dingemans et al., 2022) or frequency (Griffiths et al., 2017; Mack et al., 2010; Mito et al., 2014; Nakagawa et al., 2019; Russell et al., 2005; Ruta et al., 2010; Wikramanayake et al., 2018) between groups. In some cases, these lack of significant differences may be attributable to differences in sample characteristics across studies, including the absence of formal autism diagnoses in the comparison group (Mito et al., 2014; Wikramanayake et al., 2018) or the use of different standardized measures (i.e., Padua Inventory – Revised; Dingemans et al., 2022). In contrast, other studies reported more frequent (Bejerot et al., 2014; McDougle et al., 1995; Zandt et al., 2007) or more intense (Cadman et al., 2015) washing/cleaning compulsions by individuals with OCD or individuals with both conditions (Cadman et al., 2015; Helverschou et al., 2009; Lewin et al., 2011) compared to autistic individuals.

A limited number of studies examined differences in miscellaneous compulsions (Griffiths et al., 2017; McDougle et al., 1995; Mito et al., 2014; Nakagawa et al., 2019; Wikramanayake et al., 2018; Zandt et al., 2007) and compulsions involving others (Griffiths et al., 2017; Mack et al., 2010; Ruta et al., 2010; Zandt et al., 2007), each finding no significant differences in the frequency of these compulsions endorsed between groups. Magical/superstitious compulsions were only compared in children. Two studies reported more frequent endorsement of magical/superstitious compulsions amongst children with OCD compared to autistic children (Zandt et al., 2007) and children with both conditions (Mack et al., 2010). Griffiths et al. (2017) reported no significant differences between autistic children and children with both conditions.

Only two adult studies examined differences in self-injurious behaviors, with mixed findings. McDougle et al. (1995) found that autistic adults reported more frequent ‘self-damaging’ compulsions than adults with OCD, and Helverschou et al. (2009) found that adults with both conditions endorsed more severe self-injurious behaviors compared to autistic adults. However, differences in the measures used to assess self-injurious behavior (Y-BOCS versus Psychopathology in Autism Checklist, respectively), or the presence of an intellectual impairment in Helverschou et al. (2009)’s sample, may have contributed to the differences in these findings. Taken together, while some consistent patterns emerged in relation to the content of repetitive behaviors, there were exceptions in relation to the frequency and severity of some types of compulsions between groups.

#### Factors Associated with Repetitive Behaviors Across Groups

A number of studies investigated correlates or predictors of repetitive behavior, including associations with neurological factors, aspects of EF, social and communication factors, psychological or experiential factors, and age.

#### Neurological Factors

Several studies highlighted associations between neurological factors and repetitive behavior. Seven child studies incorporated a brain imaging component (Akkermans et al., 2019; Gooskens et al., 2019, 2021; Hollestein et al., 2021; Jacobs et al., 2021; Kushki et al., 2019; Naaijen et al., 2017), with only three reporting differences in repetitive behavior based on neurological markers. Two studies associated the intensity of repetitive behaviors with decreased glutamate concentration in autistic children, or increased glutamate concentration in autistic children and children with OCD (Hollestein et al., 2021; Naaijen et al., 2017, respectively). Akkermans et al. (2019) implicated increased functional connectivity in some brain areas with greater intensity of repetitive behaviors in autistic children and children with OCD, though between-group comparisons were not reported. Further, Hollestein et al. (2021) associated increased striatal activity (i.e., cognitive demand) during failed inhibitory control to greater compulsivity in children with OCD, suggesting an association between compulsivity and difficulties in inhibition in OCD. In contrast, Naaijen et al. (2017) found no differences in striatal activity between autistic children and children with OCD, despite significantly greater compulsivity amongst autistic children.

#### Executive Functioning

Several aspects of executive functioning (EF) were also related to the intensity of repetitive behaviors. Four studies incorporating a brain-imaging component examined differences in behavior between children with OCD versus autism (Gooskens et al., 2019, 2021), or between empirically derived transdiagnostic subgroups (Jacobs et al., 2021; Kushki et al., 2019). Due to the nature of their approach, Jacobs et al. (2021) and Kushki et al. (2019) did not report autism- and OCD-specific group comparisons. Gooskens et al. (2019) and Gooskens et al. (2021) found no associations between repetitive behavior and cognitive control assessed via a modified stop-signal task. In contrast, in an eye-tracking study, DiCriscio et al. (2016) reported a significant association between antisaccade errors and RRBIs (via the ADOS) in a group of autistic children, and in contrast no relationship between cognitive control of visual attention and the intensity of compulsions in children with OCD). This suggests a link between autism-specific repetitive behavior and cognitive control of visual attention.

Via a network analysis, Dingemans et al. (2022) posited difficulties relating to cognitive flexibility (i.e., problems with set-shifting and attention switching, and rigid cognitive style) as central and potentially perpetuating factors associated with manifestations of repetitive behavior in adults with a range of disorders (including autism and OCD). Also using a mixed sample of adults with a range of psychiatric conditions including autism and OCD, Bejerot et al. (2014) reported that greater obsessive-compulsive symptom severity was associated with poorer global functioning, though in both cases between-group differences were not reported. As well, in a sample of adults with OCD and adults with co-occurring conditions, Mito et al. (2014) found the overall obsessive-compulsive symptom severity was positively correlated with difficulties in attention switching, attention to detail, and imagination (via the AQ) in the total sample.

#### Social and Communication Factors

Several studies found that social and communication factors contributed to manifestations of repetitive behavior. In every case, these factors were compared using the Autism Spectrum Quotient (AQ; Baron-Cohen et al., 2001). In addition to cognitive inflexibility, Dingemans et al. (2022) posited difficulties related to components of social behavior (social communication, perception of social cues) as central, perpetuating factors associated with repetitive behavior in autistic adults and adults with OCD. Cath et al. (2008) reported that obsessive-compulsive symptom severity was significantly associated with social difficulties in autistic adults and adults with OCD. Pertusa et al. (2012) found that hoarding behaviors (difficulties discarding, and excessive acquisition) were positively correlated with communication difficulties amongst autistic adults, but not adults with OCD.

#### Psychological/Experiential Factors

Three studies reported on psychological or experiential factors associated with repetitive behavior, generally finding no differences in metacognitive beliefs about, or control associated with, engaging in repetitive behaviors between autistic adults and adults with OCD. Cath et al. (2008) examined ego-dystonia associated with repetitive behaviors between adults with OCD and adults with both conditions, finding no significant differences in the degree to which repetitive behaviors were experienced as excessive, unreasonable, strange, abnormal, or inappropriate, or the extent to which the person felt the need to exhibit control over their thoughts or actions. Melchior et al. (2021) examined beliefs about the necessity of performing ritual behaviors, via the Beliefs About Rituals Inventory (BARI; McNicol & Wells, 2012), also finding no significant differences between autistic adults and adults with OCD. Comparatively, Ruta et al. (2010) examined insight (i.e., individuals’ degree of awareness into the senselessness/excessiveness of beliefs related to repetitive behaviors) via a single CY-BOCS item. While neither the autistic or OCD groups displayed excellent insight into their repetitive behaviors, and ratings of good, fair, and poor insight were similar between groups, autistic individuals displayed significantly higher rates of absent insight, which was attributed to potential differences in perceiving, processing, and describing repetitive behaviors, which may indicate group differences in the experience of these behaviors.

#### Age

One study posited differences in repetitive behavior in children with OCD as a product of age. Zandt et al. (2007) found sameness behavior to be significantly more prevalent in younger children with OCD. Age was not significantly related to endorsement of sameness behaviors, repetitive motor behaviors, or compulsions in autistic children.

## Discussion

We systematically examined similarities and differences in repetitive behavior between autistic individuals and individuals with OCD, finding considerable overlap in the expression and content of repetitive behaviors using a range of measures. There were generally no significant differences between groups in the overall endorsement of autism-related RRBIs for adults, although some exceptions were noted when comparing groups of children. In a single study, Sturm et al. (2018) found that autistic children reported significantly greater endorsement of RRBIs compared to children with OCD via the SRS. This is perhaps unsurprising as the SRS is an autism measure and thus may be more sensitive to the identification of autism-specific behaviors rather than more general presentations of repetitive behavior such as those observed in OCD. Further discrepancies were noted when compulsivity (measured in the context of RRBIs) was compared via the RBS or the RBS-R, which seem to correspond with revisions made to this measure where the revised form includes more complex RRBIs such as ritualized behaviors, insistence on sameness behaviors, and restricted interests (Bodfish et al., 1999, 2000). Thus, significantly greater endorsement of RRBIs by children with OCD when assessed via the RBS-R rather than the RBS may be a result of more complex forms of repetitive behaviors now being captured and may indicate differences in the complexity of repetitive behaviors endorsed by children with OCD compared to autistic children of a similar age.

The presence of an OCD diagnosis was generally associated with significantly higher obsessive-compulsive symptom severity than in autism only. Studies which found non-significant effects predominantly compared behaviors between individuals with OCD and those with both conditions, suggesting similar symptom severity irrespective of whether OCD occurs in conjunction with autism or alone. Only one of the thirty-one included studies reported no significant differences in overall obsessive-compulsive symptom severity when comparing individuals with OCD to autistic individuals (Dingemans et al., 2022). However, this result may have been an artifact of the measure used; Dingemans et al. (2022) assessed OCD symptoms via the Padua Inventory – Revised, whereas most other studies used the CY-BOCS/Y-BOCS. Overall, these results suggest that while autism and OCD populations may be differentiated in terms of the frequency and intensity of obsessive-compulsive symptomology at a group level, they generally could not be differentiated based on characteristics related to autism with similar results at a group level.

Findings related to the content of repetitive behaviors were mixed, particularly in relation to OCD-related compulsions. The frequency of endorsement of washing/cleaning and ordering/arranging compulsions was mixed across studies, indicating that more research is needed to understand if different repetitive behavioral content is seen in each condition. In contrast, relatively consistent findings were reported for checking and hoarding compulsions. Generally, checking compulsions were more frequently and intensely endorsed by individuals with OCD. This aligns with previous research acknowledging checking compulsions as one of the most common types of compulsions manifested in OCD (Ruscio et al., 2010). The ego-dystonic nature of these compulsions may be more characteristic of the anxiety which underpins OCD, which may explain why these behaviors are more frequently endorsed by individuals with OCD than by autistic individuals (Paula-Pérez, 2013).

Autistic adults generally endorsed more hoarding compulsions compared to individuals with OCD. Previous research suggests that hoarding behaviors are common among autistic individuals (e.g., Storch et al., 2016). In a recent qualitative study, autistic adults reported that hoarding behaviors were motivated by difficulties discarding possessions, a need for emotional aids, and collecting items related to their special interests (Goldfarb et al., 2021). Thus, for autistic people, hoarding behaviors may be more often motivated by emotional attachment rather than a compulsive need or response, differentiating them from hoarding behaviors manifested in OCD. In relation to this finding, it is noted that recent changes to the diagnostic criteria view hoarding disorder as separate from OCD (APA, 2022). Regardless, hoarding behaviors were commonly included in measures of both autism- and OCD-related repetitive behavior and were hence included in the current review. Considering recent amendments to the diagnostic classification of hoarding behaviors, more research examining the distinction between these behaviors and other types of repetitive behaviors is still needed.

Findings were mixed in terms of the factors associated with repetitive behaviors. In many cases, studies reported on factors associated with an autism or OCD diagnosis, rather than correlates of repetitive behavior specifically. Further, associated factors were only reported for repetitive behaviors as a unidimensional phenomenon, so comparisons based on specific subtypes of repetitive behavior were not possible. In children, studies highlighted associations between repetitive behavior and neurological factors such as differences in glutamate concentration or striatal activity. However, only two studies examined these differences between groups. Hollestein et al. (2021) associated increased striatal activity with increased compulsivity in children with OCD, suggesting a relationship between increased cognitive demand and compulsive behavior in OCD. In contrast, Naaijen et al. (2017) found no differences in striatal activity between autistic children and children with OCD, despite significantly greater compulsivity amongst autistic children. Several previous studies have also highlighted links between striatal activity and repetitive behavior, though much of this research has been conducted using animal models (e.g., Longo et al., 2022; Muehlmann et al., 2020). To better understand the links between neurological factors and manifestations of repetitive behavior, more studies assessing repetitive behaviors as a multidimensional construct are needed. This may allow for clearer identification of the neurological features associated with different types of behavior. As well, longitudinal studies which track changes in repetitive behavior over time may be helpful for understanding how these behaviors change, and whether changes in repetitive behavior correspond with physiological, neurodevelopmental, or environmental factors.

Challenges in EF were associated with greater intensity of repetitive behaviors in autistic children. This association did not extend to children with OCD, suggesting that EF may be a factor related more closely to RRBIs in autism than to obsessive-compulsive symptomology. In support, a recent meta-analysis by Iversen and Lewis (2021) confirmed significant associations between elevated levels of RRBIs and poorer EF (including set shifting, inhibitory control, and parent-rated EF) in autistic children. Based on their findings, Iversen and Lewis (2021) surmised that impairments in EF, particularly set shifting, may contribute to the intensity of repetitive behavioral presentations, due to difficulties related to control over thoughts and behaviors.

While much of the previous literature has evaluated EF and RRBIs in younger children, similar findings relating impairments in EF to more intense RRBIs have also been found for autistic adolescents (e.g., Miller et al., 2015) and adults (e.g., Lopez et al., 2005). Two studies included in the current review (Dingemans et al., 2022; Mito et al., 2014) suggested an association between the intensity of repetitive behaviors and aspects of EF, including cognitive flexibility and attention switching. However, these associations related to individuals with a variety of obsessive-compulsive spectrum disorders (including autism and OCD), limiting the degree to which these findings can be attributed to one condition over the other. Further research investigating the role of EF on manifestations of repetitive behavior, particularly in autistic adults and/or adults with OCD including group comparisons, is warranted to understand specific mechanisms that affect each condition and better inform support or treatment.

The association between social and communicative factors and repetitive behaviors was acknowledged in several studies involving adults. While some previous research suggests connections between repetitive behaviors and social behaviors (e.g., Martínez-González et al., 2022; Rojas et al., 2006), others posit that autism-related social and communication differences are not associated with corresponding levels of RRBIs (e.g., Mandy & Skuse, 2008). Further, studies directly comparing these differences between autistic individuals and those with OCD appear limited. Thus, more research exploring the associations between social skills and corresponding engagement in RRBIs may be warranted to understand the social mechanisms that may drive transdiagnostic engagement in repetitive behaviors.

Similarities related to psychological and experiential factors when engaging in repetitive behaviors were also noted between autistic individuals, individuals with OCD, and those with both conditions. Cath et al. (2008) reported no significant differences in the ego-dystonicity of repetitive behaviors, or perceived control over repetitive behaviors, between adults with OCD and adults with both OCD and autism. This non-significant finding may be due to both groups having OCD, thus experiences associated with repetitive behaviors may have been associated with compulsions rather than with autism-related RRBIs. In contrast to this hypothesis however, Melchior et al. (2021) also found no group differences in beliefs about the necessity of performing ritualistic behaviors between autistic adults and adults with OCD. This may indicate that, irrespective of whether repetitive behaviors are experienced as ego-dystonic or ego-syntonic, autistic individuals and individuals with OCD may feel equally compelled to engage in repetitive behaviors. The reasons underpinning this drive, however, remain unclear. Differentiating between the motivating factors underpinning engagement in repetitive behaviors in future research may offer new insights into how these drivers may be different or the same in autism versus OCD and could inform supports or treatment.

Several gaps in the extant literature are highlighted by this review. In comparison to OCD-specific compulsive behaviors, contrasts between groups in relation to autism-related RRBIs were more rarely investigated and are important to further explore. Nine of the included studies examined autism characteristics via the AQ or SCQ, neither of which includes a subscale measure of RRBIs. As a result, this limited the number of studies which could offer insights into the content of RRBIs and how these varied between groups. As well, item-level comparisons based on subtypes of repetitive behavior were generally missing from the literature, particularly in relation to autism-specific RRBIs, limiting the degree to which between-group differences based on specific types of repetitive behaviors could be compared. Features associated with repetitive behaviors also tended not to be compared between groups. Instead, features were associated with diagnosis, rather than with repetitive behaviors directly, limiting the degree to which assertions about the links between these factors and repetitive behaviors could be made.

A lack of sampling diversity was also noted. From the 31 studies included in this review, it is likely that some members of the autistic and OCD communities may be underrepresented, limiting the generalizability of the results to these populations. Few studies reported on ethnicity (*n* = 4), and where reported, participants were mostly White, suggesting that other ethnicities may not be well represented in the current review. Further, in the autistic and co-occurring condition subgroups, a large proportion of participants were male (74% and 65%, respectively; Table 1), and none of the included studies examined sex or gender as a potential predictor of repetitive behavior. This is an important omission to note given the associations between sex and gender differences and different manifestations of repetitive behaviors often seen between autistic men and women (Bourson & Prevost, 2022). Further, only two studies included participants with an intellectual impairment or below average intelligence, despite individuals with intellectual differences representing as much as 38% of the autistic community (Maenner et al., 2023). Repetitive behaviors may manifest differently, and be motivated by different factors, amongst individuals with an intellectual impairment. The ego-dystonic versus ego-syntonic experience of these behaviors may be less clear than in populations without intellectual or language impairment as understanding relies more heavily on observable behaviors rather than reported experience. Underrepresentation of autistic individuals with higher support needs remains an enduring issue in the scientific literature and represents an important direction for future research to ensure that individuals with a variety of support needs are included in, and can benefit from, the findings of future research.

The results of this review should be interpreted with some limitations in mind. First, grey literature was not included, nor were non-English publications, the latter of which may have exacerbated cultural/ethnic bias. Second, while a thorough appraisal of the quality of each included study was undertaken, this review did not exclude eligible papers based on quality. While care was taken to consider the quality of each study during analysis of the results, in instances where studies did not attain adequate quality ratings, these findings must be interpreted cautiously. Alongside quality ratings, studies were also considered for input from autistic or OCD community members. Of the included studies, none acknowledged input from individuals with lived experience of repetitive behaviors in the context of autism and/or OCD, which may provide more subjective insights into differences in behavioral experiences between diagnostic groups. Future research must consider how to better integrate co-design practices and involve individuals with lived experience (in this case, autistic individuals and/or individuals with OCD) in the research process. Relatedly, while the current review was focused on quantitative studies, qualitative studies may yield differing insights into subjective experiences of repetitive behaviors, and their underlying motivations and outcomes, and are thus an important consideration in future research.

Given the significant degree of overlap in repetitive behavioral presentations observed across diagnostic groups based on standardized scores, the results of this review emphasize that accurate differential diagnosis cannot be determined, nor accurately informed by a score above a certain threshold on one or more measures, given overlapping and often non-significant group findings. Total scores on questionnaires are not sufficient to inform accurate clinical diagnosis without a thorough understanding of the factors and functions driving the repetitive behavior. While a plethora of research has identified anxiety as an inherent component of obsessive-compulsive behavioral manifestation, the links between function and behavior are not clear in the context of autism. Based on the findings of this review, OCD-specific measures of repetitive behavior (e.g., the Y-BOCS) may be more helpful than autism-specific measures in differentiating between conditions as evidence indicates that total and some subscale scores may differ at least between OCD and OCD and autism, and autism alone. Despite this, given the high degree of co-occurrence observed between autism and OCD and the evidenced overlaps in the expression and content of repetitive behaviors, it may be useful for clinicians to utilize diagnostic measures that consider both conditions in early diagnostic assessments. Furthermore, analyses of repetitive behaviors based on total scores only limit the degree to which unique behaviors can be critically compared and subsequently understood. Some types of repetitive behaviors may be more frequent or severe in autism or OCD, such as hoarding in autistic adults or magical/superstitious compulsions in children with OCD, as highlighted in the current findings. However, more research at the subscale and item-level is needed to confirm differences in the manifestation of different types of repetitive behaviors between conditions.

We highlight the ambiguity surrounding characterizations of repetitive behaviors in autism versus OCD in this review. Despite the use of similar assessment tools, findings pertaining particularly to the content of repetitive behaviors are inconsistent, suggesting more research is needed to understand to degree to which behavioral presentations across these two conditions are the same and different. At least at a phenotypical level, similarities in the expression and content of repetitive behavior are notable. Therefore, deeper understanding of the function and experience of repetitive behaviors is needed to differentiate between behavioral features in these two clinical populations more clearly. While individuals may exhibit the same behaviors, the factors driving the use of these behaviors may differ, and subsequently correspond to a distinct underlying diagnosis and importantly, different treatment and supports. In the case of co-occurring diagnosis, this may be a particularly pertinent consideration, given that these individuals may exhibit both autism-related repetitive behaviors which support self-regulation, and OCD-related compulsions manifested in response to psychological distress. Future research aimed at clearly differentiating between these experiences with repetitive behavior will inform more accurate differential diagnosis and treatment planning and maximize the benefits of therapeutic support for autistic individuals, individuals with OCD, and individuals with both conditions.
