# Correction: Repetitive Behaviors in Autism and Obsessive-Compulsive Disorder: A Systematic Review

**DOI:** 10.1007/s10803-024-06450-y

**Published:** 2024-07-02

**Authors:** Jessica O’Loghlen, Matthew McKenzie, Cathryne Lang, Jessica Paynter

**Affiliations:** 1 Building N23, -1.03, 170 Kessels Road, Nathan, QLD 4111 Australia; 2 School of Applied Psychology, Griffith University, 58 Parklands Drive, Southport, QLD 4215 Australia

**Correction to: Journal of Autism and Developmental Disorders**

10.1007/s10803-024-06357-8

In Table 1 of this article, the data in the last column headed “Autism + OCD (n = 262)” were missed out. The corrected Table 1 is given below.

**Table 1:** Demographics of autistic participants, participants with OCD, and participants with both diagnoses

Demographic		No. of studies where reported (for subgroups) *n*	Autism (*N* = 1123)	OCD (*N* = 982)	Autism + OCD (*n* = 262)
			*n* (%)		
**Age group**		31 (*n* = 2367)			
	Children		194 (17.3%)	130 (13.2%)	35 (13.4%)
	Children/adolescents		272 (24.2%)	232 (23.6%)	60 (22.9%)
	Adolescents/adults		73 (6.5%)	53 (5.4%)	0
	Adults		584 (52.0%)	567 (57.7%)	167 (63.7%)
**Gender**		30 (*n* = 2175)	1109	866†	200†
	Male		820 (73.9%)	424 (49.0%)	130 (65.0%)
	Female		289 (26.1%)	442 (51.0%)	70 (35.0%)
**Co-occurring conditions**		13 (*n* = 422)‡			
	Attention-deficit hyperactivity disorder		35 (17.6%)	11 (8.5%)	8 (8.5%)
	Anxiety		34 (17.1%)	20 (15.5%)	11 (11.7%)
	Mood disorders		50 (25.1%)	36 (27.9%)	24 (25.5%)
	Panic/agoraphobia		14 (7.0%)	6 (4.7%)	0
	Tics		3 (1.5%)	28 (21.7%)	25 (26.6%)
	Tourette’s syndrome		0	1 (0.8%)	2 (2.1%)
	Dysthymia		9 (4.5%)	2 (1.6%)	1 (1.1%)
	Social phobia		14 (7.0%)	12 (9.3%)	10 (10.6%)
	Social anxiety disorder		7 (3.5%)	1 (0.8%)	2 (2.1%)
	Specific phobia		0	5 (3.9%)	6 (6.4%)
	Separation anxiety		0	2 (1.6%)	3 (3.2%)
	Oppositional defiant disorder		11 (5.5%)	1 (0.8%)	3 (3.2%)
	Conduct disorder		1 (0.5%)	0	0
	Eating disorders		3 (1.5%)	2 (1.6%)	0
	Post-traumatic stress disorder		5 (2.5%)	0	1 (1.1%)
	Schizophrenia		4 (2.0%)	0	0
	Psychosis		9 (4.5%)	0	0

†Missing data from one study (Wikramanayake et al., 2018)

‡Autism *n* = 199; OCD *n* = 129; Autism + OCD *n* = 94

The original article has been corrected.

